# The Dollar and Blood Aristocracy

**Published:** 1880-09

**Authors:** 


More than one residence in New-York has cost, with its furnishings, not much less, exaggerations lain aside, than two hundred thousand dollars! These men have made their own money by severe industry and patient assiduity in business; and we are rather fearful that we are not a little impertinent in making any special remark about the outlay of what is their own. The fact is, we like a generous expenditure of one's means; it elevates the man, and has an elevating influence on all about him, his servants, his tradesmen, his friends, his children, and all. It is your poor, pitiful, narrow-hearted, close-fisted, mean-minded miser, who never parts with a dollar but with a pain; that is the kind of man on whom we look with unpitying contemptuousness. But for all this, we have often inquired whether any parent, wisely kind, can bring up his children in a style and manner of living which he cannot leave them the means of sustaining. There are men so stupid, that their heads cannot be turned by any elevation; no unanticipated heights make them dizzy. But to descend safely, to do it in youth, to begin married life with a declivity, who is equal to it? not one in many thousands. And what is the result? ye merchant princes, ye successful stock-jobbers, ye retired bankers of New-York, Philadelphia and Boston, we repeat the inquiry. What is the necessary result, as a general rule, as affecting the destinies of your children, who cannot, if they go out into the world, sustain the style of their father's house? The boys decline marriage, and with it give up, at one fell swoop, the purities, the joys, the elevations of domestic life. The next thing is to join some "Club," where introductions are soon made to the cigar, the wine-cup, the chess-board, the coarse jest, the loud laugh, the bacchanal song, the rail against "*Puritanism*," the Sabbath drive, or yachting, or sauntering. Then comes apace things said and done, which the pure ears of beauty can never hear, nor eyes see, nor hearts conceive, without mantling the young cheek with shame.

As for your daughters, so loving and so loved to you, what is their future? To marry "upwards," as the world calls it, they cannot. Nor can they marry men, except in rare instances, who can even *maintain* the style of living in their father's home. They must therefore marry downwards, or not marry at all, and not marrying, may almost as well be dead. In a few years, and father and mother will be gone. Brothers have formed other ties. One by one of the associates of other years is lost from their visiting list, by removal, or marriage, or death. Every year leaves them more and more lonely, more and more neglected; and soon thereafter the great world loses sight of them; their very names are only now and then mentioned, while all this time they are consuming themselves with sad memories, and anon pass unwept into a forgotten grave.

Therefore, we say to *wealthy parents, if you truly love your children, live in that style which you can enable each one of them to sustain.*

